# Supplementary material for: Global hypo-methylation in a proportion of glioblastoma enriched for an astrocytic signature is associated with increased invasion and altered immune landscape
Source: eLife. 2022 Nov 22;11:e77335. doi: 10.7554/eLife.77335 (PMC9681209; doi:10.7554/eLife.77335)
Supplement: Figure 2—source data 1. [file elife-77335-fig2-data1.zip › Figure_2_source_data_1/Figure_2C/homerResults/motif5.similar.html]

motif5

## Information for motif5

C
G
A
T
T
A
G
C
G
T
C
A
C
G
T
A
C
A
T
G
T
C
G
A
T
G
A
C
C
T
G
A
C
G
T
A
C
A
G
T
C
A
T
G
C
T
A
G
G
A
T
C
G
A
C
T
G
A
T
C
G
C
T
A
T
C
A
G
G
C
T
A
C
T
A
G
C
A
T
G
G
T
C
A
G
T
C
A
C
T
G
A
C
G
A
T
A
T
C
G
G
C
A
T
T
G
A
C
C
G
A
T
C
A
T
G
T
C
A
G
G
C
T
A
C
G
A
T
A
G
C
T
A
C
G
T
C
G
A
T
C
T
A
G
G
T
C
A
C
G
A
T
C
A
G
T
C
G
T
A
A
C
G
T
C
G
T
A
C
A
T
G
A
C
G
T
C
T
G
A
  
Reverse Opposite:  

G
A
C
T
T
G
C
A
G
T
A
C
G
C
A
T
T
G
C
A
G
C
A
T
G
T
C
A
G
C
T
A
C
A
G
T
G
A
T
C
G
C
T
A
T
G
C
A
T
C
G
A
C
G
T
A
C
G
A
T
A
G
T
C
G
T
A
C
G
C
T
A
A
C
T
G
C
G
T
A
T
A
G
C
G
C
T
A
A
G
C
T
C
A
G
T
C
A
G
T
G
T
A
C
G
A
T
C
C
G
A
T
A
G
T
C
C
G
A
T
C
T
A
G
C
G
T
A
C
T
A
G
G
A
T
C
G
T
A
C
G
T
C
A
G
C
A
T
A
G
C
T
A
C
T
G
A
G
C
T
G
T
A
C
G
C
A
T
C
A
G
T
A
T
C
G
C
G
T
A
  

|  |  |
| --- | --- |
| p-value: | 1e-20 |
| log p-value: | -4.633e+01 |
| Information Content per bp: | 1.450 |
| Number of Target Sequences with motif | 17.0 |
| Percentage of Target Sequences with motif | 1.45% |
| Number of Background Sequences with motif | 3.0 |
| Percentage of Background Sequences with motif | 0.06% |
| Average Position of motif in Targets | 117.7 +/- 47.1bp |
| Average Position of motif in Background | 98.0 +/- 23.2bp |
| Strand Bias (log2 ratio + to - strand density) | 1.0 |
| Multiplicity (# of sites on avg that occur together) | 1.06 |
| Motif File: | file (matrix) reverse opposite |

### Similar de novo motifs found

|  |  |  |  |  |  |  |  |
| --- | --- | --- | --- | --- | --- | --- | --- |
| Rank | Match Score | Redundant Motif | P-value | log P-value | % of Targets | % of Background | Motif file |
| 1 | 0.699 | G A C T A G T C G C T A C T G A T C A G T G C A T C A G C T G A T G C A C G A T C A T G A T C G G A C T C A G T T A C G C G T A T C A G C G T A C A T G C T A G G C T A C G A T G T C A C G A T C A T G C G A T G A T C G C A T A C T G T A C G G C T A G A C T G C A T A C G T C G A T | 1e-17 | -41.228811 | 1.11% | 0.03% | motif file (matrix) |
| 2 | 0.804 | G C A T A G T C G T C A G C T A C T A G C T G A G T A C C T A G G C T A A C G T C T A G T C A G G T A C C G A T G C A T C G T A C T A G C G T A C A T G C T A G G T C A G C T A C T G A C A T G C A T G A C G T G T C A C G A T C A T G T C A G C G T A C G A T A G C T C A G T C G A T C G A T G T C A G A C T G C A T G C T A A G C T G C T A C A G T A C G T C A G T C T G A C G A T G C A T G C T A C G A T | 1e-17 | -39.257095 | 1.28% | 0.05% | motif file (matrix) |
| 3 | 0.801 | C A G T A G T C G T C A C G T A A C T G G T C A A G T C T C G A G T C A G A C T T C A G T C A G G A T C C G A T G T A C C G T A T A C G C G T A C T A G C T A G G C T A T C G A C G T A C A G T A C T G G C A T G T C A G C A T A C T G T C A G G C A T A C G T A C G T A G C T A G C T A T C G G A T C G A C T G C A T G C A T | 1e-14 | -32.440788 | 1.11% | 0.04% | motif file (matrix) |
